# Clerical Employments

**Published:** 1856-12

**Authors:** 


### CLERICAL EMPLOYMENTS.

The Doctor of Divinity, who recommends clergymen to take up some other employment, as additional means of earning money for their support, has lived to little purpose, if he has not observed two simple facts, and observed them millions of times.

1st. That there never was a time, in the history of our country, when an efficient ministry—a ministry working day and night—was more pressingly needed.

2d. That to be efficient in any calling, to make every stroke tell, all the time and energies of the man, soul and body, must be concentrated on that one calling.

The Master said the laborer was worthy of his hire; the servant thinks differently.

